# Cognitive Fatigue Influences Time-On-Task during Bodyweight Resistance Training Exercise

**DOI:** 10.3389/fphys.2016.00373

**Published:** 2016-09-01

**Authors:** James R. Head, Matthew S. Tenan, Andrew J. Tweedell, Thomas F. Price, Michael E. LaFiandra, William S. Helton

**Affiliations:** ^1^U.S. Army Research Laboratory, Human Research and Engineering DirectorateAberdeen Proving Ground, MD, USA; ^2^Psychology Department, George Mason UniversityFairfax, VA, USA

**Keywords:** cognitive fatigue, bodyweight resistance training exercise, mental workload

## Abstract

Prior investigations have shown measurable performance impairments on continuous physical performance tasks when preceded by a cognitively fatiguing task. However, the effect of cognitive fatigue on bodyweight resistance training exercise task performance is unknown. In the current investigation 18 amateur athletes completed a full body exercise task preceded by either a cognitive fatiguing or control intervention. In a randomized repeated measure design, each participant completed the same exercise task preceded by a 52 min cognitively fatiguing intervention (vigilance) or control intervention (video). Data collection sessions were separated by 1 week. Participants rated the fatigue intervention with a significantly higher workload compared to the control intervention (*p* < 0.001). Additionally, participants self-reported significantly greater energetic arousal for cognitively fatiguing task (*p* = 0.02). Cognitive fatigue did not significantly impact number of repetitions completed during the exercise task (*p* = 0.77); however, when cognitively fatigued, participants had decreased percent time-on-task (57%) relative to the no fatigue condition (60%; *p* = 0.04). RPE significantly changed over time (*p* < 0.001), but failed to show significant differences between the cognitive fatigue intervention and control intervention (*p* > 0.05). There was no statistical difference for heart rate or metabolic expenditure as a function of fatigue intervention during exercise. Cognitively fatigued athletes have decreased time-on-task in bodyweight resistance training exercise tasks.

## Introduction

Similar to athletic performance, Soldier safety, and mission success are contingent on both physical and cognitive performance. Indeed, cognitive performance (e.g., decision making, psychomotor performance, response inhibition, and vigilance) plays a key role in overall Soldier performance (Friedl et al., 2007; Wilson et al., 2013). Moreover, failures in Soldiers' cognitive ability could result in increased likelihood of human error resulting in friendly-fire incidents and collateral damage (Belenky et al., 1994; Wilson et al., 2013). Though technology may enhance Soldier performance, it may also inundate Soldiers with increased sources of information to process. Unfortunately, more information for the Soldier to process may result in greater cognitive fatigue for Soldiers who have to process and relay information fluidly on the battlefield. For example, the U.S. Army is actively pursuing increasingly sophisticated sensor devices that provide situational awareness and threat detection to the Soldier on the battlefield (e.g., DARPA Squad-X project). Though this information is useful to the Soldier, it may present challenges such as mental resources depletion or bottlenecking of information processing causing cognitive fatigue (Kahneman and Tversky, 1973; Navon and Miller, 2002).

Exercise can undoubtedly elicit improvements in mental health and overall health (Biddle et al., 2000; Biddle and Mutrie, 2007; Hamer and Chida, 2008). Moreover, there is also evidence that regular exercise may act as a cognitive enhancer (Brisswalter et al., 2002; Ratey and Loehr, 2011; Chang et al., 2012) which could, for example, improve scholastic achievement (Keeley and Fox, 2009). Conversely, prior studies have provided evidence that physically demanding tasks completed prior to and during cognitive tasks result in cognitive impairment (Isaacs and Pohlman, 1991; Cian et al., 2000; Tomporowski, 2003). Moreover, when a cognitively demanding task is coupled with a physical task (dual-task), performance impairments can be observed on both the physical and cognitive components of the task (i.e., performance trade-offs; Green and Helton, 2011; Head et al., 2012). Green and Helton (2011); Green et al. (2014) suggest that these performance impairments may be the result of limited cognitive resources available to process the cognitive and physical task simultaneously. Interestingly, only recently have researchers begun to examine the effects of cognitive fatigue on subsequent performance in a physical task (Marcora et al., 2009; Pageaux et al., 2014).

Cognitive fatigue is the psychophysiological response generated by prolonged exposure to a cognitively demanding task which results in the subjective feeling of “tiredness” and “lack of energy” (Marcora et al., 2009; Pageaux et al., 2014). Cognitive fatigue has a measurable influence on physical performance and may provide a greater understanding of the causal factors affecting physical performance beyond neuromuscular fatigue (Smith et al., 2015). Marcora et al. (2009) examined the effects of participants performing a cognitively fatiguing task prior to completing a cycling task to exhaustion which resulted in participants reaching physical exhaustion sooner compared to the control condition. Pageaux et al. (2014) demonstrated that a motor response inhibition task [Stroop task; (Stroop, 1935)] impaired subsequent physical performance during a 5K treadmill time trial. Unlike Marcora and colleagues' task, participants were not pushed to exhaustion, but rather completed the treadmill task with a 6% slower completion time when preceded by the inhibition task. Cognitive fatigue has also been shown to impair physical performance in intermittent running (e.g., Yo-Yo Intermittent Recovery Test) and technical abilities such as passing and shooting in soccer (Smith et al., 2015, 2016). Furthermore, performance on continuous or prolonged intermittent exercise tasks is significantly impaired while high-intensity peak velocities were not significantly reduced (Smith et al., 2015). The amount of exercise performance impairment (2.3–17.8%; Marcora et al., 2009; Smith et al., 2015) is seemingly dependent on whether the task has a set end point (2.3–5.3%; Pageaux et al., 2014; Smith et al., 2015) or time to exhaustion/failure (17.2–17.8%; Marcora et al., 2009; Pageaux et al., 2014). However, the effects of cognitive fatigue on physical performance does not appear to be universally applicable. For example, a prior investigation found no differences in peak, critical, and estimated anaerobic work capacity on task performance as a function of cognitive fatigue in a countermovement jump task and 3 min all-out cycle test (Martin et al., 2015).

Collectively, there is mixed support for the influence of prior cognitive fatigue on subsequent physical performance. This may be partially due to the length of exercise time and also the modality (i.e., isometric vs. full body), but it is unknown if the existing paradigms can be generalized to the multitude of athletic and occupational endeavors that are high-intensity and require strength. For example, military occupations are often high-intensity but discontinuous by nature [e.g., negotiating obstacles, lifting, and climbing (Ortega et al., 1993)]. Furthermore, it is unclear whether cognitive fatigue influences perceived effort or task performance on a high intensity bodyweight resistance training exercise (strength-endurance) paradigm. Indeed, prior evidence has supported that disruptions in cognitive performance could be a function of level of intensity (e.g., *x* ≥ 70% VO_2_ max; Reilly and Smith (1986)). In other words, greater impairment may be observed by increasing the intensity of the exercise task. Thus, the present study aims to investigate the influence of cognitive fatigue on subsequent high intensity body weight resistance exercise that is self-paced and time limited. In line with previous research on continuous endurance exercise (Marcora et al., 2009; Pageaux et al., 2014; Smith et al., 2015), it is hypothesized that participants will have performance impairments (i.e., decreased repetitions completed) and modified task execution (i.e., decreased time-on-task) during the exercise task when preceded by cognitive fatigue.

## Materials and methods

### Participants

Eighteen (11 male, female 7) volunteers were recruited from local gyms located in Baltimore, Maryland, USA. Participants age ranged between 24 and 37 years (*M* = 28, *SD* = 3.8). All participants had at least 6 months experience participating in high intensity exercise routines and were free from any known illness or disease. The participants provided written informed consent in accordance with the Helsinki Accord and ethics permission was obtained from the U.S. Army Research Laboratory Institutional Research Board.

### Procedure

**Figure 1:**
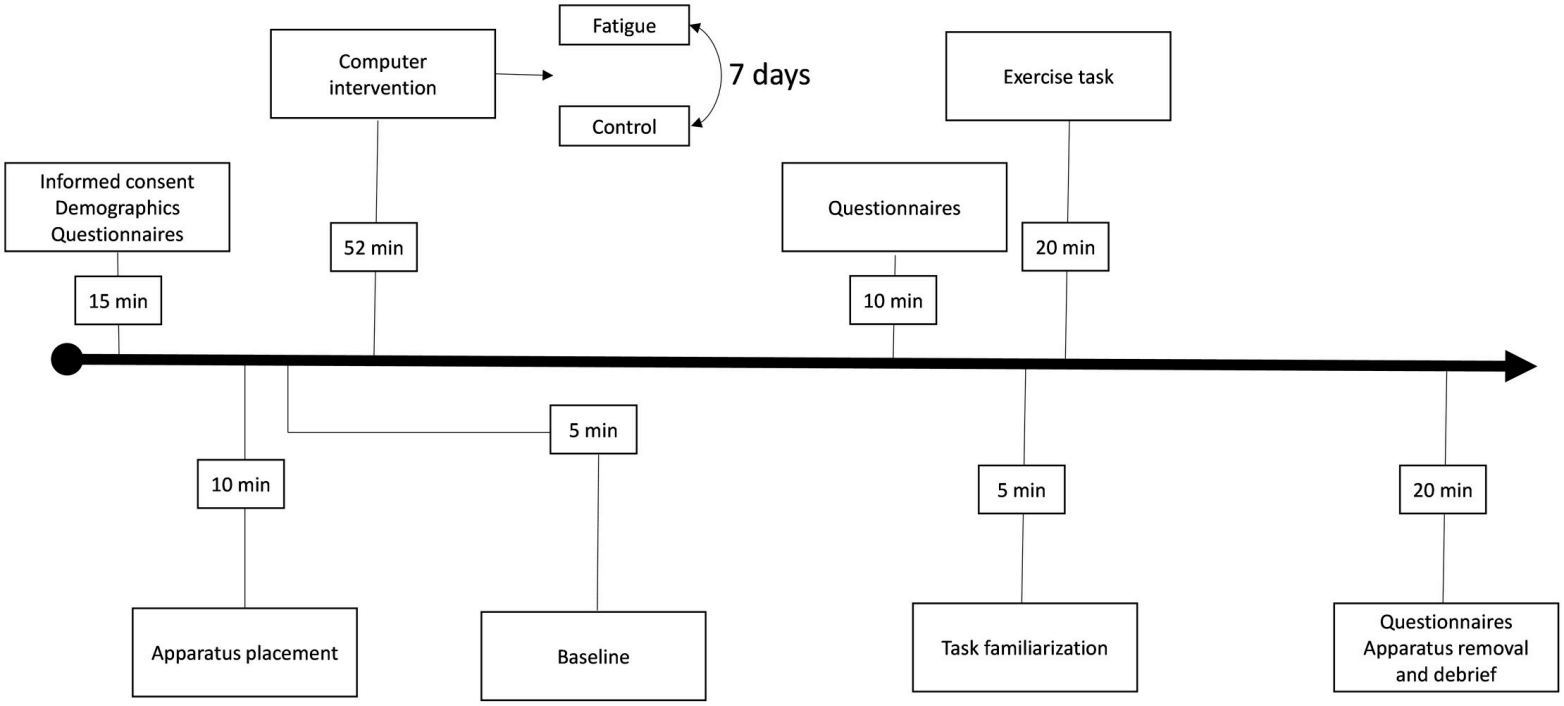
**Task timeline**. Displayed is a timeline of events with corresponding durations.

In a counterbalanced design, all participants completed either a control (passive video watching) or a cognitively fatiguing task (vigilance) prior to performing an exercise task. The two visits took place over a 2 week period (once a week) at the same time of day for each participant in an isolated room. Thus, there were approximately 7 days between sessions. Participants were given instructions to sleep for at least 7 h and were also instructed to avoid caffeine, nicotine, and other stimulants/depressants for at least 3 h before participating. Participants using stimulants and/or depressants were not permitted to participate in the study (Brownsberger et al., 2013). Upon arrival to the facility, participants completed an informed consent, demographics, and a Dundee Stress State Questionnaire (DSSQ; Matthews et al., 2002) pre-task stress state questionnaire. Within the informed consent, participants were shown a list of commonly used high-intensity interval training exercises tasks which included the exercise task used in the current investigation. Participants were requested to instruct the researchers if they there were not experienced in any of the exercise tasks. Volume of oxygen consumed (VO_2_) was used as an indirect measure of energy expenditure. Participants were outfitted with a VO_2_ measuring apparatus (COSMED K4B2, COSMED, Italy), heart rate monitor (Polar, USA) and completed a baseline VO_2_ measure whereby they sat quietly in an isolated room for 5 min. Upon completion of the baseline VO_2_ measure, participants completed the experimental or control intervention (i.e., cognitive fatigue or video watching, respectively). After the experimental or control intervention, the participant completed a National Aeronautical and Space Administration Task Load Index (NASA-TLX; Hart and Staveland, 1988) and DSSQ post-task stress state questionnaire. Prior to beginning the exercise task each time, participants completed a standardized dynamic warmup for 5 min which included familiarization with the exercise task with the VO_2_ apparatus. After completing the warmup, participants completed the exercise task for 20 min. Participants were not permitted time keeping devices and were generally unaware of the time elapsed during the 20 min exercise task. During the exercise task, participants rated their RPE every 5 min using a RPE scale (Borg, 1998; Gearhart et al., 2001). Upon completion of the exercise task, participants completed the NASA-TLX and DSSQ post-task stress state questionnaire (see Figure 1 for timeline of study).

### Cognitive fatigue intervention

A traditional vigilance task was used to induce cognitive fatigue (Warm et al., 2008). The vigilance task took place in an isolated room. Participants were seated 50 cm in front of a video display terminal (53.4 × 33 cm, 60 Hz refresh rate) which was mounted at eye level. Participants' head movements were not restrained. Time-keeping devices such as watches and cell phones were surrendered at the start of the task. Participants completed a vigilance task (low Go/ high No-Go) monitoring for numeric stimuli (1–9). Participants were instructed to respond to the number 3 and withhold their response to 1–9 except the number 3. Go stimuli were presented 11% of the time. Participants were instructed to respond as fast and accurately as possible to the target number 3. Prior to the start of the task, participants completed a practice trial where they received feedback on their performance. The vigilance task was 52 min in duration and was comprised of 8 periods of watch, 6.5 min in length. The task was continuous with no rest breaks. The inclusion of the task epochs allows the researcher to examine performance impairment (i.e., accuracy and response time) as a function of time-on-task which is a behavioral indicator of cognitive fatigue (Head and Helton, 2012). Numeric digits were all the same font (Courier); however, font size varied between 48, 72, 94, 100, and 120, with height varying between 12 and 29 mm. Varied font size was used to discourage participants from using visual shape cues (Robertson et al., 1997). Each trial consisted of a single digit presented centrally on the screen for 250 ms followed immediately by a 900 ms mask. The mask consisted of a circle (29 mm in diameter) with a diagonal line in the middle spanning from one side to the other. Participants were instructed to respond with their index finger on their dominant hand using a response box.

### Control intervention

The computer screen from the vigilance task was used to present a 52 min video train documentary. The documentary was “The American Orient Express” (Pegasus-Eagle Rock Entertainment, 2004) which consisted of footage about trains and travel. This type of stimuli has been used in similar studies due to the neutral content maintaining stable mood and heart rate (Silvestrini and Gendolla, 2007; Marcora et al., 2009; Smith et al., 2015).

### Physical exercise task protocol

The physical task used in the present study was a high intensity body resistance exercise routine. It was comprised of three separate exercise sets completed in succession for as many rounds as possible in 20 min. The first exercise was 5 pull-ups, followed by 10 pushups, and finally 15 unweighted squats. Participants were required to finish each set for each exercise prior to beginning a new round. A research investigator verbally counted completed repetitions during the exercise task. Upon the completion of each round (5 pull-ups, 10 pushups, 15 unweighted squats), the next round was immediately started. This specific exercise task was chosen because these bodyweight exercises are commonly configured together in a circuit workout at the local gym facilities where recruitment took place. Thus, participants were more likely to be experienced in the individual exercises and completing them consecutively.

All participants were given instructions regarding proper form for each exercise and inappropriate repetitions were not counted. For the pull ups, participants were instructed to begin by grasping a metal pullup bar overhead with both hands facing palm out with feet not touching the ground. Participants were instructed to pull themselves up until their chin was above the bar and then to lower themselves until their arms were fully extended. For the pushups, participants were instructed to start in a standard pushup position (plank) and then lower themselves until their chest made contact with the ground. Once their chest made contact with the floor, participants were instructed to extend their arms until they were back in the plank position. For the unweighted squats, participants were instructed to lower themselves to 90° of knee flexion (i.e., seated position). Once participants achieved the seated positon, they were instructed to return to a standing position. Participants were notified when they correctly completed each repetition by stating the number of correct repetitions performed. Additionally, participants were notified if they did not complete the exercise task correctly by saying “no repetition.” In total, only two participants were given verbal “no repetition” warnings during the pull up portion of the exercise task. Participants were not given verbal encouragement during any task. A high-definition 60 Hz frame rate video camera (Vixia HFR42, Canon, U.S.A., Inc) was placed in a standardized location ~15 m from the workout area to record the workout completed by the participant. The workout videos were later used for behavioral analysis.

### Video workout protocol

A researcher, blinded to study hypothesis and subject intervention, viewed each video and quantified the number of repetitions and rounds completed, time-on-task and time off-task. Time-on-task was defined as participants actively performing the exercise task. Conversely, time off-task was defined as participants not actively performing the exercise task (e.g., transition between exercise type and resting). The researcher was given a predefined workout protocol that provided examples of how each exercise was to be performed to be counted as a repetition. To test interrater reliability, an additional blinded research assistant viewed and evaluated a subset (22 videos) of 36 videos. An intraclass correlation of 0.90 (95% confidence interval: 0.78–0.96) revealed acceptable interrater reliability (Hays and Revicki, 2005).

### Psychological scales

#### Consciousness and stress state questionnaire

An abridged version of the 90-item DSSQ (Matthews et al., 2002) was used to measure participants subjective stress state. The abridged DSSQ contains 32-items which generate a 4-factor solution: energetic arousal (EA), task arousal (TA), task-related thoughts (TRT), and task-unrelated thoughts (TUTS). These factors have been used in prior studies to determine whether participants were focused on task related or unrelated thoughts during the task and also arousal level (Head and Helton, 2012, 2013). Items from each factor are aggregated to yield a single score for each factor for pre- and post-task. The abridged DSSQ was given prior to the experimental intervention, post-intervention, and after the exercise task.

#### Workload measure scale

The NASA-TLX is a workload measure composed of 6-items (Hart and Staveland, 1988). The questionnaire contains three items that measure external demand (mental, temporal, and physical) and an additional three items (effort, performance, and frustration) that measure internal responses to the external demands. The 6-items were aggregated together for a composite global workload score. The NASA-TLX was given after each computer task and exercise task.

#### Rating of perceived exertion

The Borg rating of perceived exertion (RPE) scale is a 15-point scale ranging from 6 “No exertion” to 20 “Maximal exertion.” The RPE scale is versatile and can be applied to aerobic and anaerobic exercise (Borg, 1998; Gearhart et al., 2001). During the exercise task, participants were requested to indicate their RPE at 5 min intervals by pointing to a numerical value on a standardized 15-point RPE poster board (54 × 50 cm) placed next to the exercise area. Participants were instructed to rate their effort expended on the exercise task. Each participant was provided verbal examples of “No exertion” (e.g., sitting on the couch relaxing) and “Maximal exertion” (e.g., you are giving all your effort).

### Statistical analysis

All data reported is presented as mean ± *SD* unless otherwise stated. A one-way repeated measures ANOVA was used to test whether response time and accuracy errors changed over time in the vigilance task as a manipulation check. Repeated measures *t*-tests were used to test whether the fatigue intervention vs. the control intervention effected percent time-on-task and exercise repetitions completed. Mean heart rate (bpm) and relative VO_2_ (mL·kg^−1^·min^−1^) were calculated for each condition (baseline, intervention, and exercise task). Heart rate and VO_2_ were subjected to separate 2 (intervention type) × 3 (baseline, intervention, and exercise task) repeated measures ANOVAs to examine whether the fatigue intervention had an effect on time periods of measurement. Mean heart rate and VO_2_ were calculated per 5 min period for the 20 min exercise tasks preceded by the fatigue and control intervention. Mean heart rate and VO_2_ were subjected to two separate repeated measures 2 (intervention type) × 4 (time period) ANOVA to determine whether heart rate and VO_2_ changed as a function of intervention type and time period. Similarly, heart rate and VO_2_ was calculated per 13 min time period for the 52 min fatigue and control intervention. Mean heart rate and mean VO_2_ per time period was subjected to two separate repeated measures 2 (intervention type) × 4 (time period) ANOVA as a manipulation check to determine whether heart rate and VO_2_ measures changed as a function of intervention type and time period. RPE was subjected to a repeated measure 2 (fatigue type) × 4 (time block) ANOVA to determine whether the fatigue intervention vs. the control intervention affected self-reported RPE as a function of time (5 min blocks). The subjective post-intervention DSSQ sub-scales for Energetic Arousal, Task Arousal, Task-Related Thoughts, and Task Unrelated Thoughts were calculated for each individual. Due to all measures being on the same response scale (i.e., 1–5) the raw (non-standardized) scores were used as recommended (Rogosa, 1995). These scores were analyzed with 2 (fatigue type) × 3 (time period: baseline, post computer task, and exercise task) × 4 (scale: EA, TA, TRT, TUT) repeated measures ANOVA. To determine perceived workload of each task, a global workload score was calculated for each participant by averaging the 6 subscales of the NASA-TLX for each participant for each task. Global workload scores were subjected to a 2 (intervention type) × 2 (task type: computer vs. exercise task) repeated measures ANOVA. When appropriate, *post-hoc* comparisons were made with the Bonferroni multiple comparison procedure. Effect sizes for repeated measures were calculated as partial eta squared (η^2^_*p*_). Effect sizes for paired *t*-test were calculated as Cohen *d*_*z*_. Significance was set at 0.05 (2-tailed). All analysis were conducted using the Statistical Package for Social Sciences, version 22 (SPSS Inc., Chicago, IL, USA).

## Results

### Manipulation check

**Figure 2:**
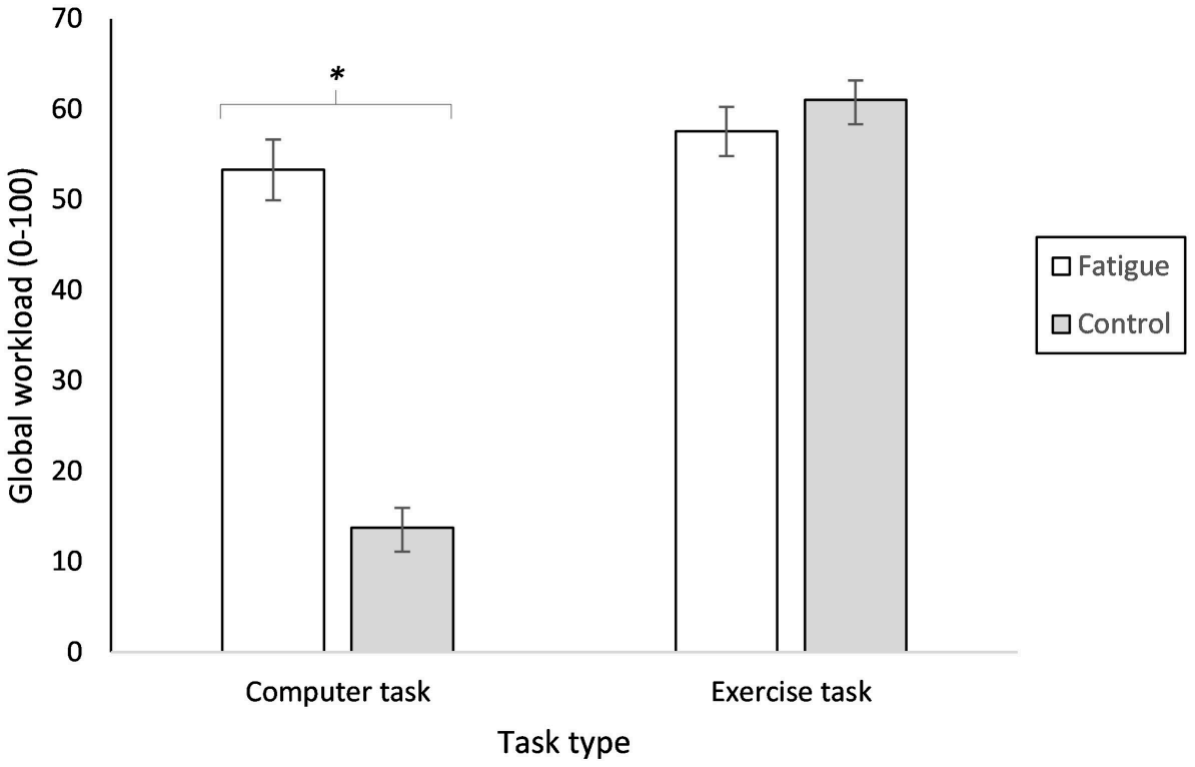
**Workload Measures**. Global workload measures for computer and exercise tasks. ^*^Significant interaction between task and intervention type (*p* < 0.001). Data are presented as mean ± SEM.

The results for correct response times to targets showed impaired performance (slowing) across the vigilance task *F*_(7, 119)_ = 27.54, *p* < 0.001, η^2^_*p*_ = 0.62. However, the result of the accuracy analysis failed to reach significance due a high accuracy rate, *F*_(1, 17)_ = 0.43, *p* = 0.520, η^2^_*p*_ = 0.07 see Table 1 for results of each time period. The response time result provided evidence that the vigilance task elicited cognitive fatigue. Prior investigations have examined relative elevated HR as a physiological marker for cognitive fatigue (Marcora et al., 2009). Thus, we examined change over time for heart rate while participants completed the cognitive fatigue and control intervention to verify whether the cognitive fatigue intervention was fatiguing. Additionally, we also analyzed whether metabolic expenditure changed as a function of time and task. For the 52 min control and fatigue intervention, there was a significant main effect of time on heart rate, *F*_(3, 51)_ = 39.32, *p* < 0.001, η^2^_*p*_ = 0.70; however, there was no main effect for intervention type, *F*_(1, 17)_ = 1.68, *p* = 0.212, η^2^_*p*_ = 0.09, or time by intervention type interaction, *F*_(3, 51)_ = 2.17, *p* = 0.103, η^2^_*p*_ = 0.11. There was a main effect for time period for VO_2_, *F*_(3, 51)_ = 8.97, *p* < 0.001, η^2^_*p*_ = 0.35, but no main effect for intervention type, *F*_(1, 17)_ = 0.23, *p* = 0.636, η^2^_*p*_ = 0.01, or time by intervention type interaction, *F*_(3, 51)_ = 0.82, *p* = 0.489, η^2^_*p*_ = 0.05. Global workload, as measured by the NASA-TLX, revealed significant main effects for intervention type, *F*_(1, 17)_ = 82.80, *p* < 0.001, η^2^_*p*_ = 0.83, and task type, *F*_(1, 17)_ = 94.59, *p* < 0.001, η^2^_*p*_ = 0.85. Moreover, there was a significant intervention type by task type interaction, *F*_(1, 17)_ = 70.83, *p* < 0.001, η^2^_*p*_ = 0.81. Overall, the exercise task was rated with significantly higher workload measures relative to the intervention and control task (see Figure 2).

**Table 1:** **Descriptive statistics (M; ***SD***) for each time period of the cognitive task**.

**Period**	**1**	**2**	**3**	**4**	**5**	**6**	**7**	**8**
Response time ms	358 (37.33)	392 (46.24)	407 (56.00)	416 (56.00)	417 (60.67)	426 (61.09)	428 (64.06)	439 (69.58)
Accuracy %	99.1 (2.12)	99.7 (0.89)	99.7 (0.89)	99.2 (1.87)	99.1 (1.65)	99.4 (1.19)	98.4 (2.54)	99.5 (1.06)

### Physical performance and task execution

**Figure 3:**
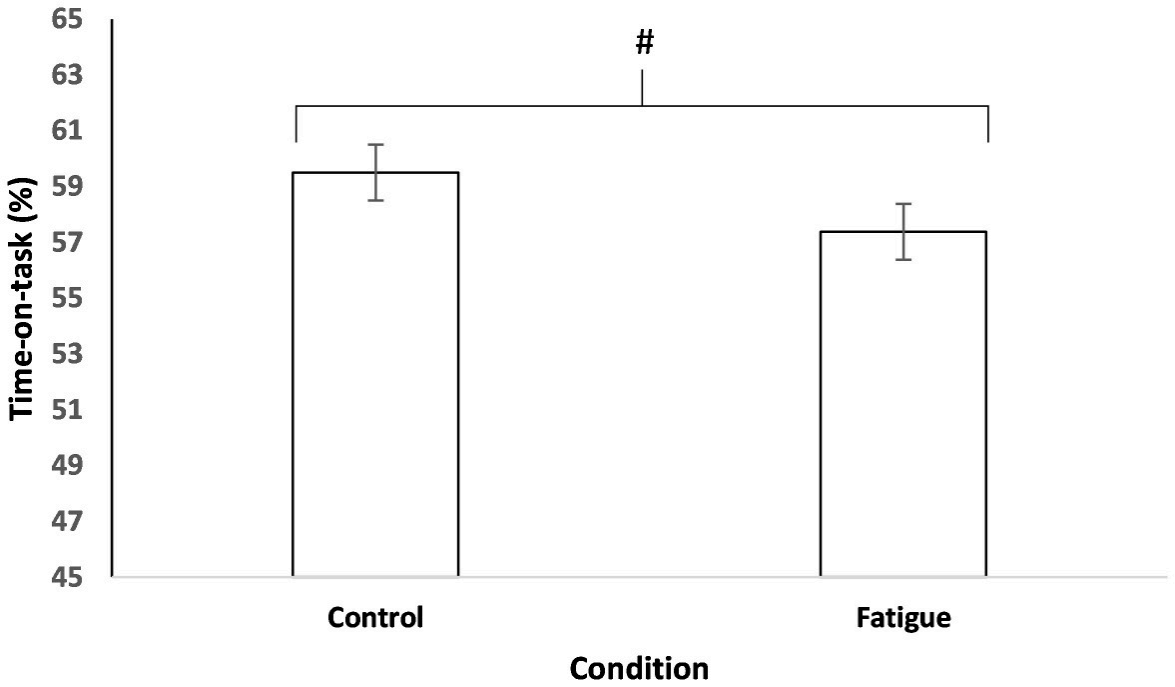
**Task execution**. Percent time-on-task completed as a function of cognitive fatigue or control. ^#^Significant main effect for condition (*p* < 0.05). Data are presented as mean ± SEM.

**Figure 4:**
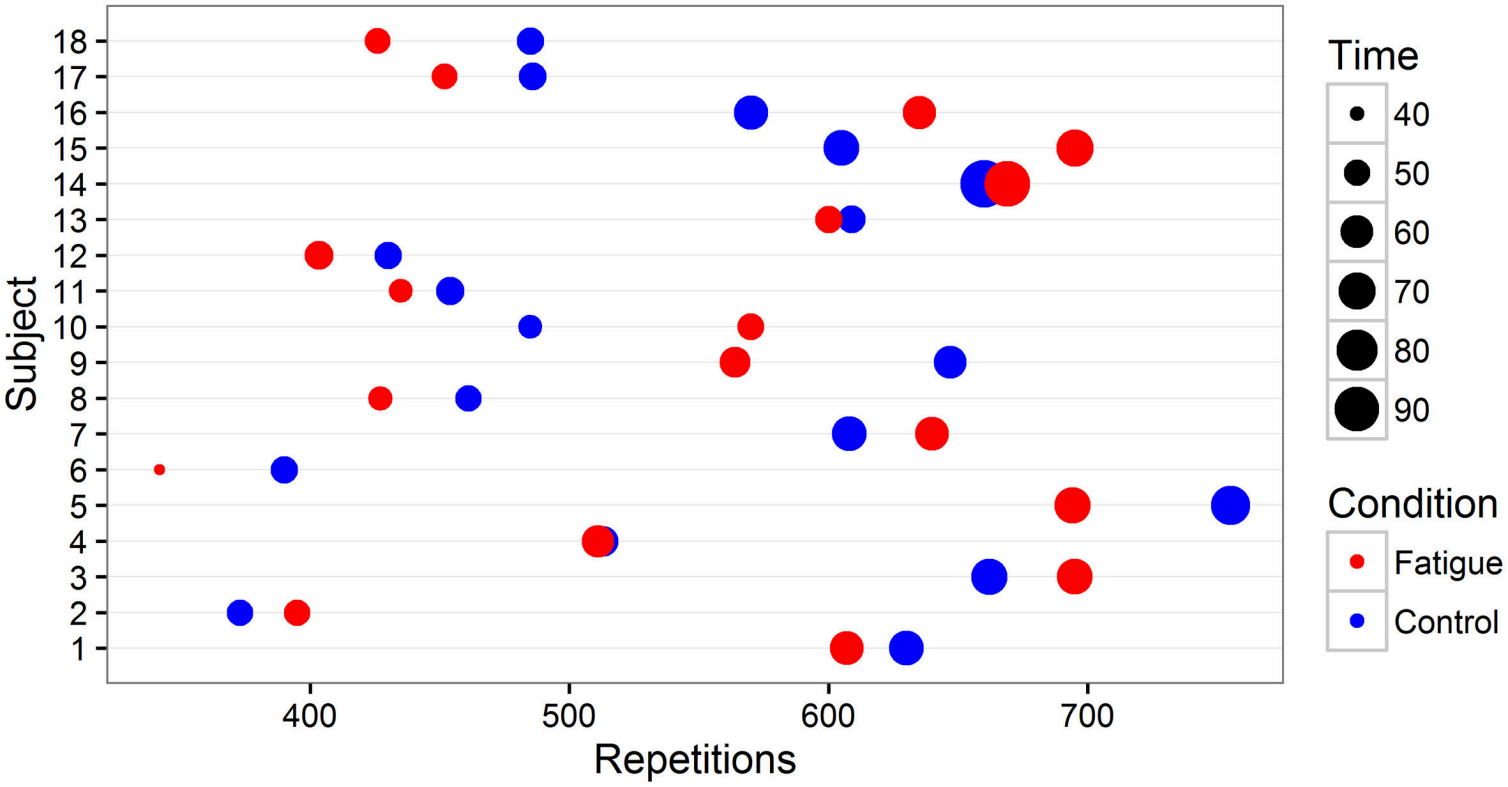
**Individual performance and task execution**. Each participant (*n* = 18) is displayed on the y-axis. Percent time-on-task is visually expressed as magnitude of icons. Position of fatigue and control icons on the x-axis correspond to repetitions completed as a function of intervention.

Participants completing the cognitively fatiguing intervention showed no significant difference in repetitions between the fatigue intervention (542; *SD* = 119.10) relative to the control intervention (545; *SD* = 107.05), *t*_(17)_ = 0.30, *p* = 0.772, *d*_*z*_ = 0.07. In total, 11 of the 18 participants completed less repetitions per time when completing the cognitive fatigue intervention relative to the control prior to the exercise task. Participants completing the fatigue intervention spent significantly less time-on-task (57%; *SD* = 12.43) relative to the control intervention (60%; *SD* = 11.88), see Figure 3, *t*_(17)_ = 2.26, *p* = 0.037, *d*_*z*_ = 0.53. For individual performance, including percent time-on-task and repetitions can be see Figure 4.

### Physiologic measures

There was a significant main effect of time for heart rate and VO_2_ [*F*_(2, 34)_ = 246.06, *p* < 0.001, η^2^_*p*_ = 0.94; *F*_(2, 34)_ = 992.44, *p* < 0.001, η^2^_*p*_ = 0.98, respectively]. All other main effects and interactions failed to reach significance *p* > 0.05. *Post-hoc t*-test with Bonferroni corrections for time periods are presented in Table 2. Regardless of intervention or control, participants only had significant increases in heart rate and VO_2_ when comparing the intervention and exercise task measures. For each 20 min exercise task preceded by the control and fatigue intervention, there was a significant main effect of time period for heart rate, *F*_(2, 34)_ = 246.06, *p* < 0.001, η^2^_*p*_ = 0.94; however, there was no main effect for intervention type, *F*_(1, 17)_ = 0.67, *p* = 0.425, η^2^_*p*_ = 0.04, or time by intervention type interaction, *F*_(2, 34)_ = 0.81, *p* = 0.452, η^2^_*p*_ = 0.05. There was a main effect for time period for VO_2_, *F*_(2, 34)_ = 992.44, *p* < 0.001, η^2^_*p*_ = 0.98, but no main effect for intervention type, *F*_(1, 17)_ = 0.009, *p* = 0.928, η^2^_*p*_ = 0.001, or time by intervention type interaction, *F*_(2, 34)_ = 0.49, *p* = 0.933, η^2^_*p*_ = 0.004.

**Table 2:** **Descriptive statistics (M; ***SD***) and significance test for time period and fatigue type**.

	**Cognitive fatigue**			**Control**		
	**Pre-CT**	**CT**	**ET**	**Pre-CT**	**CT**	**ET**
Heart rate	51.8 (30.10)	70.6 (8.90)	159.9 (19.50)^$^^†^^***^	57.5 (30.95)	67.7 (9.32)	164.5 (10.60)^$^^†^^***^
VO_2_ ml·kg^−1^ min^−1^	3.9 (2.54)	4.1 (4.24)^$^^*^	32.9 (19.93)^$^^†^^***^	4.1 (.14)	4.3 (0.42)	32.7 (22.90)^$^^†^^***^

*CT, computer task; HR (bpm), heart rate; ET, exercise task*.

$ *Main effect for time period*.

† *significantly different from CT*.

*** corresponds to p < 0.001;

* *p < 0.01*.

### Psychological measures

**Figure 5:**
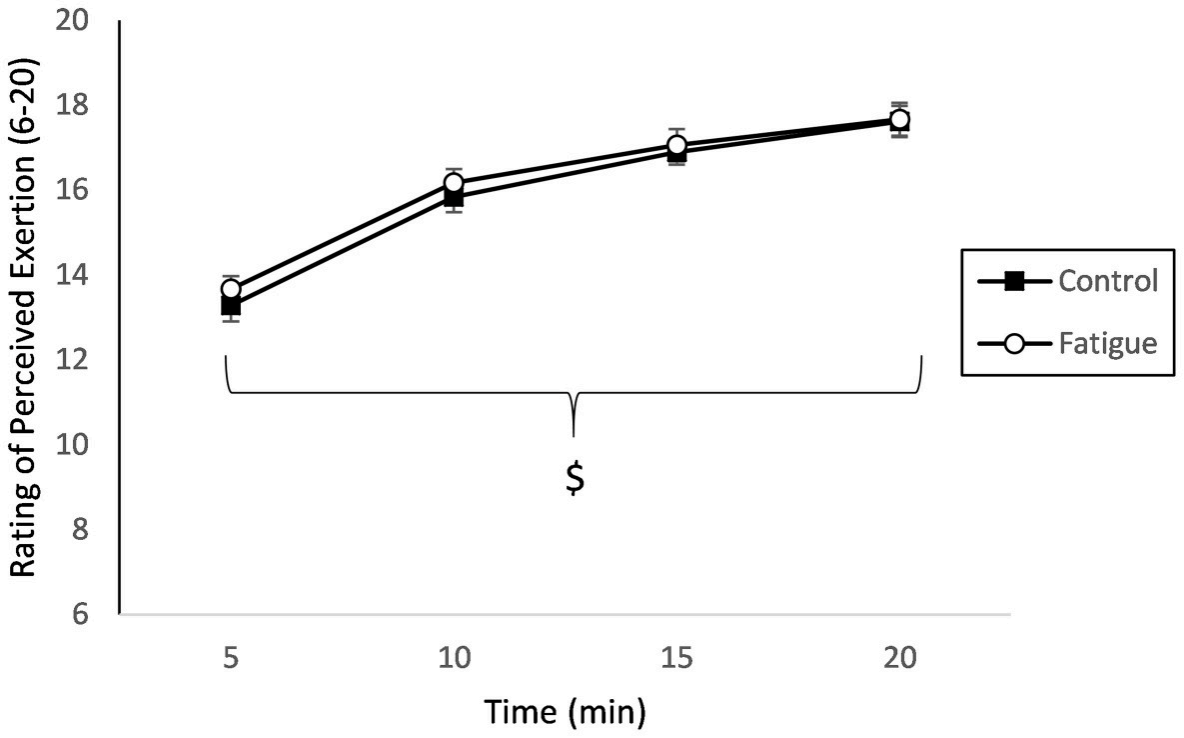
**Perceived Exertion**. RPE as a function of time-on-task raw scores. ^$^indicated main effect of time (*p* < 0.001). Data are presented as mean ± SEM.

RPE revealed a main effect of time, *F*_(1, 51)_ = 153.53, *p* < 0.001, η^2^_*p*_ = 0.90. All other main effects (*p* = 0.45, η^2^_*p*_ = 0.006) and interactions (*p* = 0.67, η^2^_*p*_ = 0.002) failed to reach significance (see Figure 5). The subjective post-intervention DSSQ analysis revealed a 3-way interaction between intervention type, time period, and scales, *F*_(6, 102)_ = 2.59, *p* = 0.022, η^2^_*p*_ = 0.13. Bonferroni *post-hoc* tests revealed that for intervention there was a significant difference between fatigue and control intervention for energetic arousal and task related thoughts (*p* = 0.019). Additionally, there were differences between baseline and the fatigue intervention (*p* = 0.019) and exercise task and fatigue intervention (*p* = 0.009). See Table 3 for a complete list of DSSQ results.

Table 3**Descriptive statistics (M, ***SD***) and significance test for the DSSQ scales and conditions**.

**Table:** 

	**Mental fatigue**			**Control**		
	**Pre-CT**	**Post-CT**	**Post-ET**	**Pre-CT**	**Post-CT**	**Post-ET**
EA	16.61 (1.87)	16.50 (1.95)	17.11 (2.29)	16.00 (2.46)	14.22 (2.67)	16.67 (2.84)
TA	15.67 (1.82)	16.05 (2.96)	12.17 (2.29)	15.39 (2.16)	15.11 (3.30)	12.83 (3.18)
TRT	15.78 (4.67)	22.67 (5.09)	19.06 (3.01)	13.89 (3.90)	16.17 (5.09)	19.61 (5.52)
TUT	13.00 (3.48)	15.17 (4.67)	9.72 (4.07)	12.06 (3.31)	13.28 (6.36)	10.94 (3.27)

**Table:** 

**Effect**	**DF**	***F***	***P***	η^2^*p*
Fatigue	(1,17)	6.77	0.019	0.29
Time point	(2,34)	7.47	0.002	0.31
Scale	(3,51)	32.5	<0.001	0.66
Fatigue × time point	(2,34)	7.23	0.002	0.30
Fatigue × scale	(3,51)	3.74	0.017	0.18
Time point × scale	(6,102)	13.77	<0.001	0.45

*CT, computer task; ET, exercise task; EA, Energetic Arousal; TA, Tense Arousal; TRT, Task Related Thoughts; TUT, Task Unrelated Thoughts*.

## Discussion

This study examined the influence of prior cognitive fatigue on subsequent physical performance of a high intensity full body resistance exercise task. The cognitive fatigue intervention was subjectively difficult and did produce cognitive fatigue for participants. The current findings support the hypothesis that task execution (time spent performing the task) on a full body resistance exercise task can be negatively impacted when preceded by a cognitive fatiguing task.

### Vigilance induced cognitive fatigue

The vigilance decrement can be characterized as an increased proportion of missed targets and slower response rate to targets over time (Mackworth, 1948; Head et al., 2012; Head and Helton, 2012, 2013). Individually and in combination, these behavioral measures reflect the difficulty and cognitively fatiguing nature of vigilance tasks (Warm and Jerison, 1984). The vigilance task utilized in the current investigation failed to show physiological differences in heart rate or VO_2._

The vigilance intervention was successful in eliciting cognitive fatigue. Participants had slowed response times to targets across the vigilance intervention which is a behavioral indicator of cognitive fatigue (Warm and Jerison, 1984). The high accuracy rate coupled with the elevated task related thoughts suggests that participants were focused on the task. Moreover, participants rated the cognitively fatiguing task as being more energetically arousing and having greater global workload relative to video control task. This elevated energetic arousal is indicative of mental resource recruitment (Warm et al., 2008; Ossowski et al., 2011). Elicited cognitive fatigue is not task specific to response inhibition or a more complex cognitive task such as the AX-CPT used in previous studies (Marcora et al., 2009; Pageaux et al., 2014). Indeed, a relatively more unadulterated measure of sustained attention such as the vigilance task used in the current study had measureable effects on physical activity.

### Effects of cognitive fatigue on full body resistance exercise

Importantly, the percent time-on-task measure was sensitive enough to show statistical differences. Participants who were cognitively fatigued showed a 3% decrease in time-on-task relative to the no fatigue condition. Repetitions completed was possibly too coarse of a measure, lacking the sensitivity to show a performance difference between the fatigue and control conditions. Indeed, a *post-hoc* power analysis revealed that the repetitions measure (*1* − β = 0.06) had far less power relative to the percent time-on-task measure (*1* − β = 0.58). Importantly, the results provide evidence that physical engagement with an exercise task can be influenced by prior cognitive fatigue. In other words, participants might be more prone to engage in rest breaks during a physical task when previously cognitively fatigued.

### Effects of cognitive fatigue on physiological measures During full body resistance exercise

The current results demonstrated that the respective baseline conditions did not significantly differ from either the cognitively fatiguing task or video control task with concern to VO_2_ and heart rate. Moreover, the cognitively fatiguing and control task did not significantly differ from each other with respect to VO_2_ and heart rate. As expected, there was increased metabolic expenditure and heart rate in exercise tasks relative to the baseline and computer task. As found in prior investigations, the results did not show significant differences in heart rate and metabolic expenditure as a function of cognitive fatigue (Marcora et al., 2009; Pageaux et al., 2014). Had the number of repetitions been substantially different between the interventions, an increase in both mean VO_2_ and heart rate would be expected. It is likely that the measure of repetition count, VO_2_ and heart rate are all too coarse to observe the 3% difference observed in time-on-task (Darter et al., 2013).

### Psychological effects of cognitive fatigue

The workload measure, as indexed by NASA-TLX, provided corroborating evidence that the vigilance intervention was cognitively fatiguing relative to control. Moreover, the energetic arousal measure was elevated after the vigilance task relative to the control task indicating that participants were actively engaged with the task. This is further supported by the high accuracy rate during the vigilance task. In other words, the vigilance task was cognitively demanding and participants were focused on the task. The task related thoughts measure provided evidence that participants had a significant increase in task related thoughts upon completing the vigilance task; however, task related thoughts decreased significantly after completion of the exercise task. This result may suggest a shifting focus from the extrinsic goal of completing the task to unrelated stimuli (Helton et al., 2011; Ossowski et al., 2011). As suggested by the lower time-on-task after cognitive fatigue, participants may be less willing to provide maximal effort when cognitively fatigued (Marcora et al., 2009; Pageaux et al., 2014; Smith et al., 2015, 2016).

### Limitations and directions for future research

The current investigation provides evidence that cognitive fatigue impairs time on task during a high intensity bodyweight resistance exercise task. However, some limitations are present and should be identified. Though the current investigation and prior studies on this topic have utilized different time lengths (30–90 min), cognitively fatiguing task types (e.g., response inhibition and vigilance) and stimuli (i.e., alphanumeric), it is unknown whether the difficulty of the cognitively fatiguing task has differential effects on subsequent physical performance. Indeed, dual-task paradigms (i.e., completing two tasks at the same time) have provided evidence that stimuli requiring more cognitive effort to process will generate greater performance impairments on a secondary cognitive task (Head et al., 2012; Head and Helton, 2012). This same principle may hold true when a cognitively fatiguing task is performed prior to the physical task.

Given the current and prior investigations are interested in the interaction between cognitive fatigue and physical performance, future investigations may benefit from incorporating non-invasive neurologic measures of executive function (e.g., fNIRS; Head and Helton, 2013; Byun et al., 2014). For example, fNIRS can be used to estimate the level of cognitive fatigue during the cognitively fatiguing task and the subsequent physical task. Incorporating neuro-correlate measures may permit an enhanced objective understanding of the level of cognitive fatigue experienced by the participant and more importantly how it effects their physical performance.

Lastly, given the emphasis of military application, it is unknown whether Soldiers would respond similarly as their civilian counterparts. For example, Soldiers are commonly equipped with tactical gear (e.g., improved outer tactical vest, ammunition, Army combat uniform, and weapon) that is difficult to maneuver in and is often heavy. Additionally, Soldiers likely receive more physical and mental toughness training relative to their average civilian counterpart. Thus, the cognitive fatigue intervention may need to be more extreme to illicit fatigue from Soldiers relative to civilians.

### Perspective

The current findings provide evidence that cognitive fatigue impacts how subsequent physical activity is performed. Participants who are cognitively fatigued spend significantly less time-on-task (i.e., more rest breaks) than when not previously cognitively fatigued. More importantly, our findings show that the effect of cognitive fatigue is independent of specific intervention techniques (vigilance or response inhibition) and is not dependent on the exercise task being performed (running, cycling, and discontinuous exercise task). Given the evidence of impaired performance on a physical task as a function of cognitive fatigue, we advise that athletes and Soldiers do not engage in unnecessary cognitively demanding tasks prior to competitions or missions, respectively. Additionally, as suggested by Smith et al. (2015), coaches or commanders may need to assess their personnel's level of cognitive fatigue prior to physical activity.

The results of the current investigation have implications for athletes, but also for Soldiers. For example, if Soldiers engage in a cognitively fatiguing task (e.g., guard duty at a checkpoint) prior to completing a high intensity physical engagement with enemy combatants, then it may be more difficult for Soldiers to stay on task which could be detrimental due to the demanding nature of war. Additionally, performance impairments as a result of cognitive fatigue may extend beyond gross motor skills to tasks requiring fine motor actions (Duncan et al., 2015); for example, marksmanship performance. This may have direct and dire implications for Soldiers who are cognitively fatigued and then required to engage in a firefight (e.g., friendly fire). Future studies on this topic should include fine motor tasks relevant to Soldiers (e.g., marksmanship) to determine whether the effect of cognitive fatigue on subsequent physical performance is only a gross motor phenomena.

## Author contributions

JH: substantially contributed to the conception, design, acquisition and analysis of data, and interpretation of the work, was involved in drafting the work, approved the final version to be published and agree to be accountable for all aspects of the work. MT: highly contributed to the conception, design, acquisition and analysis of data, and interpretation of the work, was involved in drafting the work, approved the final version to be published and agree to be accountable for all aspects of the work. TP: highly contributed to revise the work critically for important intellectual content, approved the final version to be published and agrees to be accountable for all aspects of the work. AT: highly contributed to the conception, design, acquisition and analysis of data, and interpretation of the work, was involved in drafting the work, approved the final version to be published and agree to be accountable for all aspects of the work. ML: highly contributed to the conception, design, and interpretation of the work, was involved in drafting the work, approved the final version to be published and agrees to be accountable for all aspects of the work. WH: highly contributed to the conception, design, and interpretation of the work, was involved in drafting the work, approved the final version to be published and agrees to be accountable for all aspects of the work.

### Conflict of interest statement

The authors declare that the research was conducted in the absence of any commercial or financial relationships that could be construed as a potential conflict of interest.

## Abbreviations

ANOVA: Analysis of Variance
DSSQ: Dundee Stress State Questionnaire
EA: Energetic Arousal
NASA-TLX: National Aeronautical and Space Administration Task Load Index
RPE: Rating of Perceived Exertion
TA: Task Arousal
TRT: Task-Related Thoughts
TUT: Task-Unrelated Thoughts
VO_2_: Oxygen consumption.
